# Voltage-gated sodium channel intron polymorphism and four mutations comprise six haplotypes in an *Aedes aegypti* population in Taiwan

**DOI:** 10.1371/journal.pntd.0007291

**Published:** 2019-03-29

**Authors:** Han-Hsuan Chung, I-Cheng Cheng, Yen-Chi Chen, Cheo Lin, Takashi Tomita, Hwa-Jen Teng

**Affiliations:** 1 Center for Diagnostics and Vaccine Development, Centers for Disease Control, Ministry of Health and Welfare, Taipei, Taiwan; 2 National Mosquito-Borne Diseases Control Research Center, National Health Research Institutes, Miaoli, Taiwan; 3 Department of Medical Entomology, National Institute of Infectious Diseases, Shinjuku-ku, Tokyo, Japan; Saudi Ministry of Health, SAUDI ARABIA

## Abstract

**Background:**

Knockdown resistance (kdr) to dichlorodiphenyltrichloroethane (DDT) and pyrethroids is known to link amino acid substitutions in the voltage-gated sodium channel (VGSC) in *Aedes aegypti*. Dengue fever primarily transmitted by *Ae*. *aegypti* is an annual public health issue in Taiwan. Accordingly, pyrethroid insecticides have been heavily used for decades to control mosquito populations in the summer and autumn. In Taiwan, an *Ae*. *aegypti* population with two VGSC mutations, V1016G and D1763Y, was described previously.

**Methodology/Principal finding:**

*Aedes aegypti* (G0) were collected in Tainan and Kaohsiung in southern Taiwan. The VGSC gene polymorphisms of the *kdr* mutations and the intron flanked by exons 20 and 21 were verified. The first generation offspring (G1) were used to measure the resistance level to cypermethrin, a pyrethroid insecticide currently used in Taiwan. In addition to V1016G and D1763Y, we describe two new mutations, S989P and F1534C, which have not been reported in Taiwan. Moreover, we also identify two types (groups A and B) of introns between exons 20 and 21. Intriguingly, the *kdr* mutations S989P, V1016G and D1763Y are strictly located on the haplotype harboring the group A intron, whereas F1534C links to the group B intron. When those data were taken together, we proposed the following six haplotypes for VGSC genes in Taiwan today: (i)S989-intron A-V1016-F1534-D1763, (ii)S989-intron A-V1016G-F1534-D1763, (iii)S989P-intron A-V1016G-F1534-D1763, (iv)S989-intron A-V1016G-F1534-D1763Y, (v)S989-intron B-V1016-F1534-D1763 and (vi)S989-intron B-V1016-F1534C-D1763. Triple heterozygous mutations of either S989P/V1016G/F1534C or V1016G/F1534C/D1763Y can be found in one single *Ae*. *aegypti* mosquito. The proportions of the VGSC mutations were relevant to cypermethrin resistance. Notably, the presence of S989P and V1016G in the population could be a helpful reference to predict the resistance level to cypermethrin. This is the first study to demonstrate the coexistence of four *kdr* mutations in a population of *Ae*. *aegypti*.

**Conclusions/Significance:**

Four *kdr* mutations (S989P, V1016G, F1534C and D1763Y) and two intron forms (Group A and B) were commonly found in local *Ae*. *aegypti* populations in Taiwan.

## Author summary

VGSC mutations of *Aedes aegypti* threaten vector control programs and have been brought to attention in dengue endemic areas. Taiwan has suffered dengue outbreaks, which usually begin from an imported case. Pyrethroid insecticides were used to kill infectious females and adults in the surrounding environment of each suspected case. In Taiwan, V1016G and F1763Y mutations of VGSC have been described previously. Here, we further describe two additional amino acid substitutions (S989P, F1534C) and two forms of the intron between exon 20 and 21. We also propose six haplotypes for VGSC genes in Taiwan today. In conclusion, four *kdr* mutations (S989P, V1016G, F1534C and D1763Y) and two intron forms (Group A and B) are commonly found in local *Ae*. *aegypti* populations in Taiwan.

## Introduction

The yellow fever mosquito, *Aedes aegypti* (L.), is a metamorphic dipteran species capable of spreading chikungunya virus, dengue virus, Rift Valley fever virus, yellow fever virus and Zika virus via feeding on human blood. Its larval and pupal stages are aquatic and rely heavily on anthropogenic containers [1]. This species originated in Africa [2]. Through human activities such as transportation and urbanization in new areas, *Ae*. *aegypti* is today found in tropical and subtropical regions throughout the world [3]. In Taiwan, yellow fever mosquito habitats are strictly distributed over the southern area and Penghu (a group of islands at west side of Taiwan), whereas Asian tiger mosquitoes, *Aedes albopictus*, can be found throughout Taiwan, from sea level to 1,760 m [4]. Dengue fever contributes annually as a public health burden in Taiwan. There were 15,492 indigenous cases in 2014, and in 2015, the case number hit a record high of 43,419. The identity of geographical distribution between *Ae*. *aegypti* habitat and most indigenous cases strongly suggests that *Ae*. *aegypti* is the primary vector of dengue fever in Taiwan, whereas occasionally rare indigenous cases occurring elsewhere point out a secondary role of *Ae*. *albopictus* in the epidemiology of dengue fever [5].

For the control of mosquito-borne diseases, community engagement for habitat management and the use of insecticides are currently used. Undoubtedly, habitat management is a reliable and promising approach to lower mosquito population number with almost no disadvantages. However, the application of insecticides is a quicker method, particularly during the action to deal with imported cases and outbreaks of mosquito-borne diseases. The long-term use of insecticides promotes the development of resistance in mosquitoes. This issue is considered one of the hardest obstacles to mosquito control throughout the world [6, 7]. Among various insecticides, dichlorodiphenyltrichloroethane (DDT) and pyrethroid compounds primarily target voltage-gated sodium channel (VGSC), namely the voltage sensitive sodium channel (VSSC) (reviewed by [8]). The resistance of insects to DDT and pyrethroid is linked to knockdown resistance (kdr) [8–10]. Several amino acid substitutions in *Ae*. *aegypti* VGSC were functionally confirmed to be associated with kdr by using expressed VGSC protein in *Xenopus* oocytes [11, 12]. I1011M, V1016G and F1534C were reported to confer increasing resistance to pyrethroid compounds. S989P alone did not alter the resistance level of the recombinant protein [11, 12]. When coexpressed with V1016G, S989P was shown to enhance V1016G-mediated resistance to deltamethrin [12].

Saavedra-Rodriguez *et al*. reported that an ~250 bp intron separates codons 1011 (exon 20) and 1016 (exon 21) in the *Ae*. *aegypti* VGSC genomic region [13]. Afterward, Martins *et al*. then described a polymorphism of that intron. Based on their length, they were classified into two groups, A (250 bp) and B (234 bp) [14]. The variance in intron polymorphism could serve as a marker to study the origins of mutations in the VGSC gene. I1011M was found to coexist with the group A intron [14]. Subsequently, V1016I was reported to locate at the haplotype harboring the group A intron [15]. In other studies, the mutation F1534C was found in alleles possessing either the group A or group B intron, but apparently had a strong link to the group A intron [16].

After malaria eradication in Taiwan was certified by the World Health Organization (WHO) [17], dengue fever replaced malaria to become the most serious mosquito-borne disease in Taiwan. There are hundreds to thousands indigenous cases annually in Taiwan. For instance, 200–2000 indigenous cases of dengue fever were reported each year from 2004–2013 according to Taiwan CDC’s surveillance data (http://www.cdc.gov.tw/rwd/). To reduce the damage caused by dengue fever, pyrethroid insecticides have been used for decades. A surveillance study from 2002–2012 reported that the *Ae*. *aegypti* population in Taiwan displayed resistance to various pyrethroid insecticides [18]. In 2009, Chang *et al*. reported two VGSC mutations, V1016G and D1763Y (referred to as V1023G and D1794Y in [19], respectively), from a permethrin resistant strain, originally collected in Kaohsiung in 1990 [19]. In 2013, a study found that the two mutations were present in *Ae*. *aegypti* collected in the field from Tainan and Kaohsiung [20]. During 2014–2015, Taiwan suffered severe damage from dengue fever, mainly in Tainan and Kaohsiung: 15,492 and 43,419 indigenous cases were reported in 2014 and 2015, respectively. The failure in the fight against dengue fever during those two years might be due to an inability to efficiently control the vector, which possibly had developed new *kdr* mutations in the population. Hence, it became very important to be aware of the current VGSC gene status in *Ae*. *aegypti* population in Taiwan. In this study, we investigated the VGSC gene information for *Ae*. *aegypti* collected in 2016 in the high-risk areas of Tainan and Kaohsiung. To investigate the VGSC gene, we focused on the two previously reported amino acid sites of *kdr* mutations, V1016 and D1763, along with the other *kdr* sites with functional confirmation, including S989, I1011 and F1534. Moreover, we also characterized the polymorphic status of the intron between exon 20 and 21, in order to further clarify the relationship among those *kdr* mutations. Bioassays to examine the resistance to cypermethrin, a pyrethroid insecticide currently used in Taiwan, were carried out as well. The links between VGSC gene traits and cypermethrin resistance are also discussed.

## Methods

### Mosquitoes

**Fig 1:**
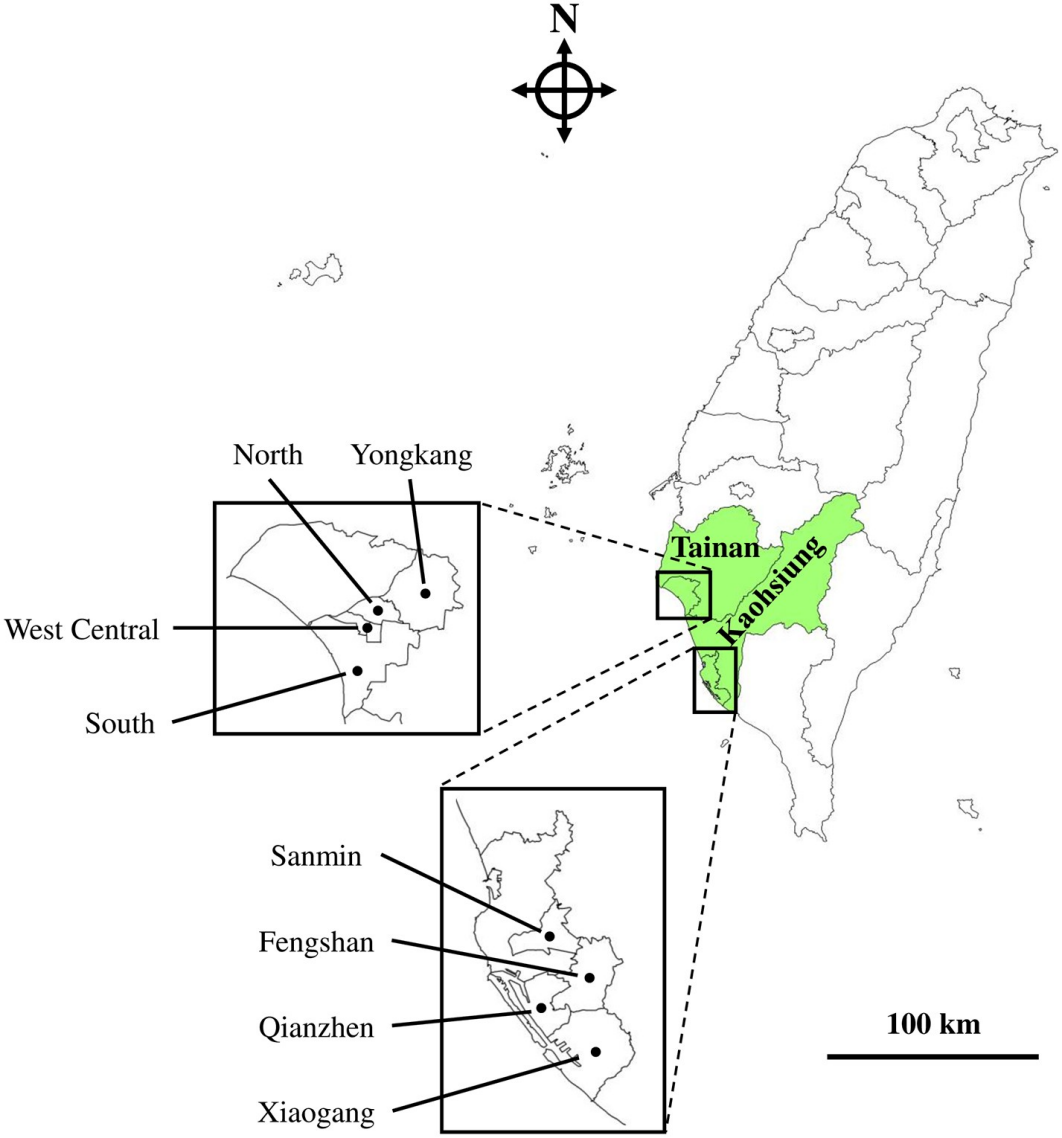
Taiwan map. Mosquito collection sites in Tainan (West Central District, South District, Yongkang District and North District) and Kaohsiung (Sanmin District, Xiaogang District, Qianzhen District and Fengshan District) are marked. The map was made by ArcGIS (https://www.arcgis.com/).

We drove along Tainan (West Central District, South District, Yongkang District and North District) and Kaohsiung (Sanmin District, Xiaogang District, Qianzhen District and Fengshan District) in southern Taiwan (Fig 1) to collect *Ae*. *aegypti* larvae and pupae in March and October 2016. Since dengue fever cases are frequently reported during the summer and autumn in Taiwan, a large amount of pyrethroid insecticides is used during that period. Therefore, we selected March and October for mosquito collection in order to verify the impact of insecticide use. Mosquito collection was carried out in public areas or private residences/lands with residents’/owners’ permission. The mosquito larvae and pupae were identified under the microscope. Species belonging to *Ae*. *aegypti* were brought back to the laboratory and reared supplied with sufficient amount of food [yeast extract/pig liver powder; 1:3 (w/w)] daily in an insectary at 20–30°C. Adults were maintained in an acrylic cage (30 × 30 × 30 cm; MegaView Science, Taichung, Taiwan) and were provided with a 10% sucrose solution. Males of the parental generation (G0) were used for VGSC gene sequencing. Eggs were collected and reared to the next generation. Females of the first generation (G1) were collected for insecticide bioassay.

### Determination of VGSC mutations and intron polymorphisms

To avoid male sperm DNA contamination in female mosquitoes, we selected only males to verify the VGSC information. The sex determination factor in *Ae*. *aegypti* is located on the first chromosome [21]. Since the VGSC gene is mapped on the third chromosome [22], theoretically, the results derived from male DNA should not cause sexual bias. Each mosquito was placed in a 1.5 ml Eppendorf tube with 80 μl phosphate-buffered saline and one glass bead (diameter 2.5 mm). Samples were homogenized using a TissueLyser (Qiagen, Hilden, Germany) while shaking for 30 sec 3 times. The supernatant was processed using a QIAamp DNA Mini Kit (Qiagen) according to the procedure supplied with the kit. Finally, the genomic DNA was eluted in 80 μl Tris-EDTA buffer for immediate use and stored at -20 °C.

**Fig 2:**
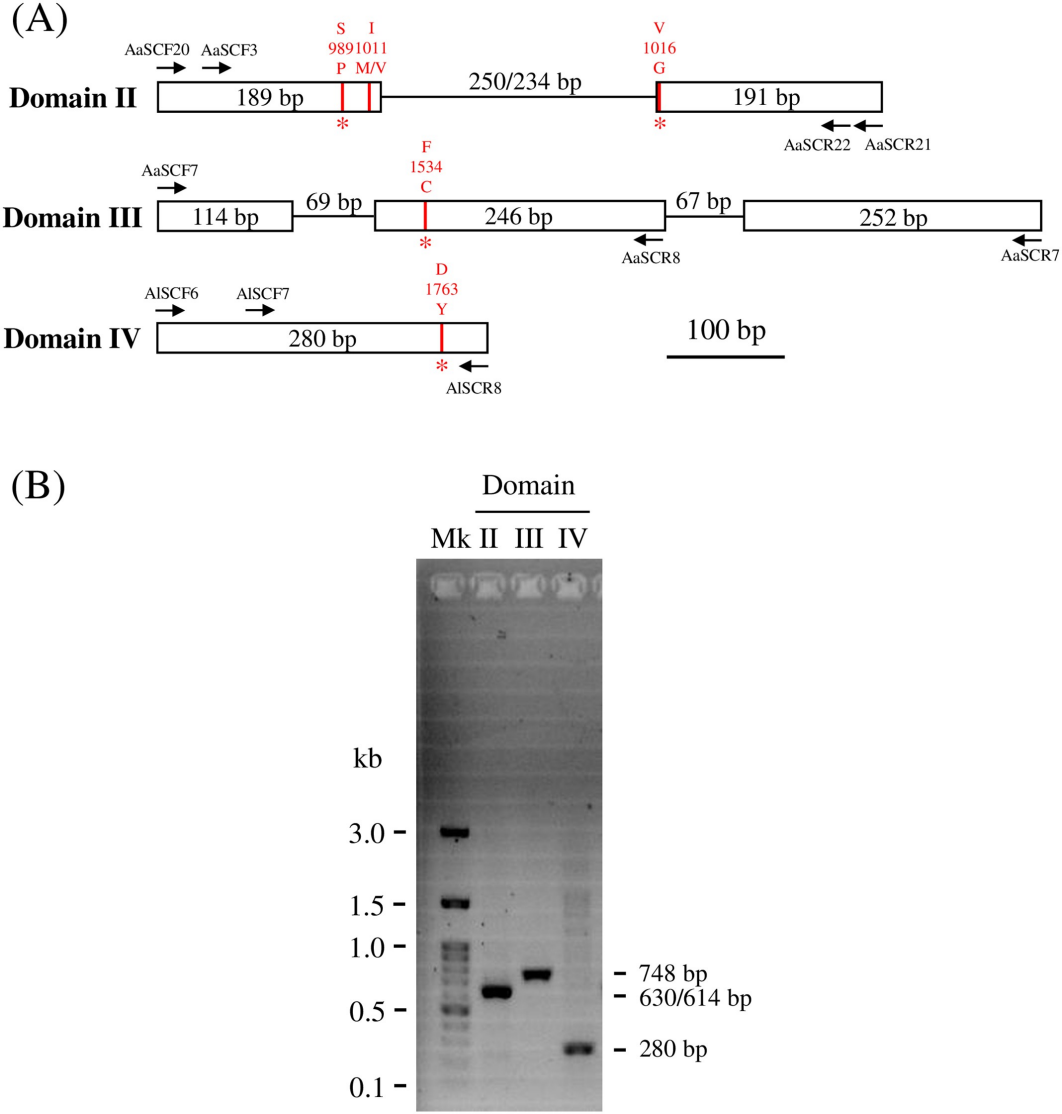
Strategies and methods for genotyping *Aedes aegypti* VGSC mutations. (A) In the partial genomic region encoding VGSC, the positions of the *kdr* mutations (red columns) and primers (arrows) are indicated. Boxes and lines represent exons and introns. The intron flanked by exons 20 and 21 is polymorphic, as its length can be 250 or 234 bp. The four *kdr* mutations S989P, V1016G, F1534C and D1763Y found in Taiwanese *Ae*. aegypti are marked by asterisks. The scale bar represents a length of 100 bp. (B) PCR products were examined using agarose gel electrophoresis. The amplicon length of the VGSC domain II is either 630 or 614 bp, due to intron polymorphism.

Sequences of primers to detect VGSC point mutations and intron polymorphisms are based on a previous study [23]. Briefly, the 630/614 bp [the length variance depends on the intron polymorphism (250/234 bp)] segment at domain II was amplified with the primers AaSCF20 (5’-GACAATGTGGATCGCTTCCC-3’) and AaSCR21 (5’-GCAATCTGGCTTGTTAACTTG-3’) and then sequenced with either AaSCF3 (5’-GTGGAACTTCACCGACTTCA-3’) or AaSCR22 (5’-TTCACGAACTTGAGCGCGTTG-3’); the 748 bp segment at domain III was amplified with primers AaSCF7 (5’-GAGAACTCGCCGATGAACTT-3’) and AaSCR7 (5’-GACGACGAAATCGAACAGGT-3’) and then sequenced with AaSCR8 (5’-TAGCTTTCAGCGGCTTCTTC-3’); and the 280 bp segment at domain IV was amplified with the primers AlSCF6 (5’-TCGAGAAGTACTTCGTGTCG-3’) and AlSCR8 (5’-AACAGCAGGATCATGCTCTG-3’) and then sequenced with AlSCF7 (5’-AGGTATCCGAACGTTGCTGT-3’). The locations of the *kdr* mutations and primers are illustrated in Fig 2A. The polymerase chain reaction was carried out using i-pfu DNA polymerase (iNtRON Biotechnology, Seongnam, Korea) at a 55 °C annealing temperature and 1 min elongation time. The amplicons were visualized by ethidium bromide staining after electrophoresis in 2% agarose gels (Fig 2B) and sent out for direct sequencing. The sequences were aligned and analyzed using GeneStudio (http://genestudio.com/).

### Insecticide bioassay with cypermethrin

For measuring the resistance to cypermethrin in mosquitoes from different districts, three-to-five-day-old G1 female adult mosquitoes were transferred in web cages (25 × 11 × 11 cm, 25 insects per cage) from acrylic cages. During the transfer, the mosquitoes were constantly supplied with a 10% sucrose solution. For each treatment, eleven cages of females (275 mosquitoes) were required. Ten cages of mosquitoes (250) were evenly hung in an ~30 m^3^ room (eight corners and two at top-middle), while one cage (25) was left behind as the untreated control. Different concentrations of 60 ml diluted cypermethrin solution were sprayed into the room by an ultra-low volume fogger. After cypermethrin treatment, the mosquitoes were kept in the room full of cypermethrin air particles for 30 min and then were moved into a collecting chamber (BioQuip Products, Inc., Rancho Dominguez, CA, USA) supplied with a 10% sucrose solution. The mosquitoes were then held in a growth chamber at 28±2°C and 75±10% relative humidity (RH) with the photoperiod of 10:14 (L:D) for 24 h. The mortality was calculated according to the criteria whether the mosquito can stand (with six or less legs) or not. Finally, the LC99 in each group was calculated with Microsoft Excel based on treated cypermethrin concentrations.

### Statistical analysis

The correlation of LC99 to either proportion of *kdr* mutations or VGSC haplotypes was examined using Pearson’s correlation coefficient model for the estimation of r^2^ value and Student’s *t*-test with Microsoft Excel.

## Results

### VGSC mutations

**Fig 3:**
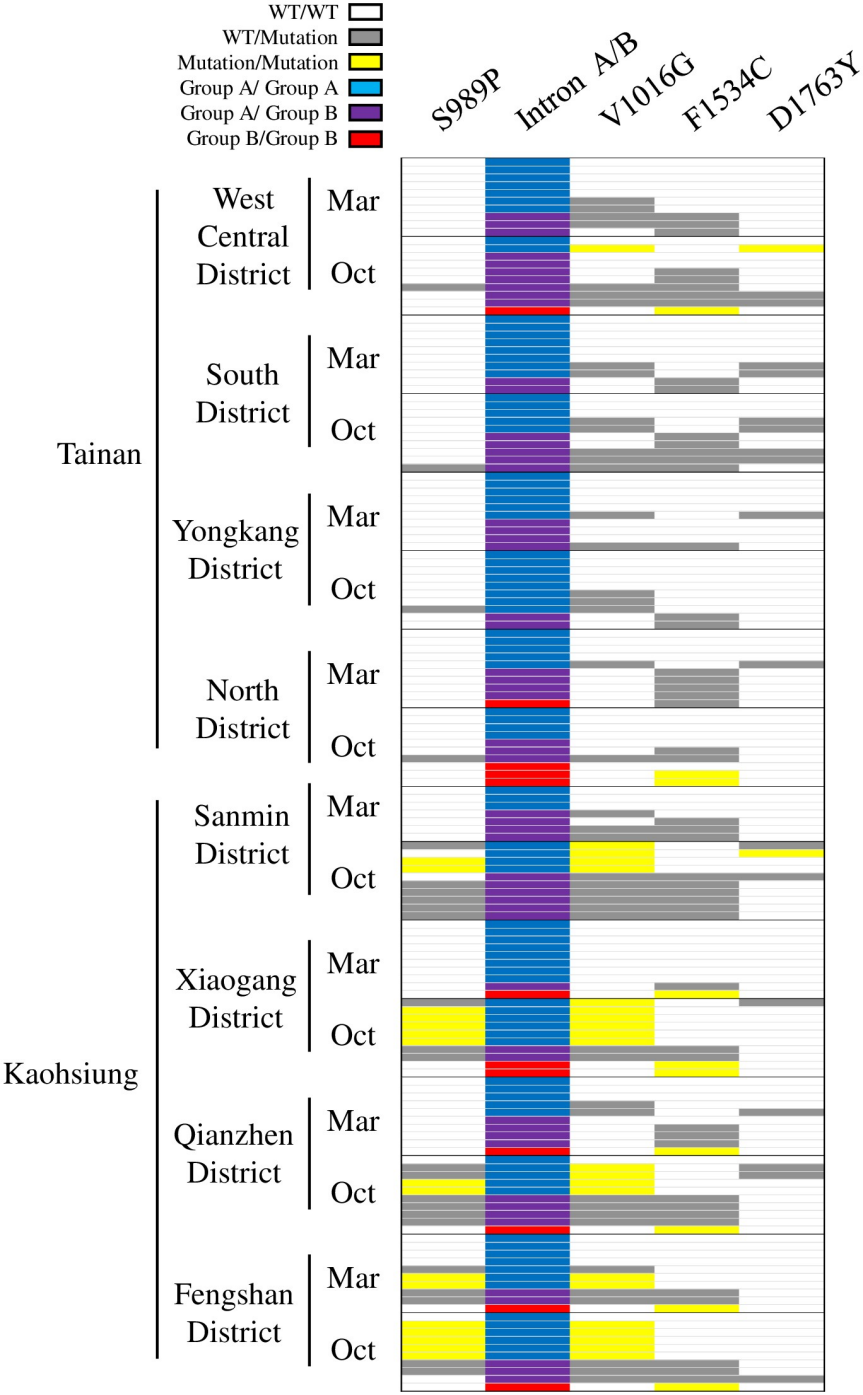
Genotypes of 157 *Aedes aegypti* collected in southern Taiwan. In the boxes illustrating *kdr* mutations, white, gray and yellow boxes represent homozygous wild type (WT/WT), heterozygous mutation (WT/Mutation) and homozygous mutation (Mutation/Mutation), respectively. For the intron polymorphism, the blue, purple and red boxes represent group A intron homozygotes (Group A/Group A), group A/B intron heterozygotes (Group A/Group B) and group B intron homozygotes (Group B/Group B).

We analyzed five VGSC mutation sites, S989P, I1011M/V, V1016G/I, F1534C and D1763Y, of the *Ae*. *aegypti* VGSC gene [the amino acid positions are numbered based on the house fly (*Musca domestica*) VGSC protein sequence]. In 157 mosquitoes collected in southern Taiwan, we observed four mutation types, namely S989P (TCC to CCC), V1016G (GTA to GGA), F1534C (TTC to TGC) and D1763Y (GAC to TAC). The four amino acid mutations resulted from DNA point mutations. The mutations I1011M/V nor V1016I were not detected (Fig 3). The four mutations were all found in eight districts from either Tainan or Kaohsiung. The most frequent mutation is V1016G (28.03%) and the rarest is D1763Y (6.69%); the proportions of S989P and F1534C are 17.83% and 21.97%, respectively (Table 1). Furthermore, V1016G can occur independently and can also accompany S989P or D1763Y. In contrast, in the presence of S989P or D1763Y, we always found V1016G (Fig 3). In the mosquitoes carrying F1534C in both alleles (F1534C/F1534C), there is no coexistence of any other mutation (S989P, V1016G or D1763Y) (Fig 3, ten F1534C/F1534C individuals among 157 mosquitoes). In both Tainan and Kaohsiung, the mosquitoes collected in October possessed more mutations of all four mutation types than those collected in March (Table 1). This phenomenon may be due to the frequent use of pyrethroid insecticides for mosquito control during the summer and autumn in Taiwan.

**Table 1:** Proportion of four *kdr* mutations and two intron types in *Aedes aegypti* collected in southern Taiwan.

Location	Month	Point mutation sites				Intron polymorphism	
		S989P	V1016G	F1534C	D1763Y	A	B
Tainan	Mar	0.00%	11.25%	13.75%	5.00%	81.25%	18.75%
		(0/80)	(9/80)	(11/80)	(4/80)	(65/80)	(15/80)
	Oct	5.00%	17.50%	25.00%	10.00%	68.75%	31.25%
		(4/80)	(14/80)	(20/80)	(8/80)	(55/80)	(25/80)
Kaohsiung	Mar	9.46%	16.22%	20.27%	1.35%	77.03%	22.97%
		(7/74)	(12/74)	(15/74)	(1/74)	(57/74)	(17/74)
	Oct	56.25%	66.25%	28.75%	10.00%	71.25%	28.75%
		(45/80)	(53/80)	(23/80)	(8/80)	(57/80)	(23/80)
Total		17.83%	28.03%	21.97%	6.69%	74.52%	25.48%
		(56/314)	(88/314)	(69/314)	(21/314)	(234/314)	(80/314)

### VGSC intron polymorphism and its link to mutations

**Fig 4:**
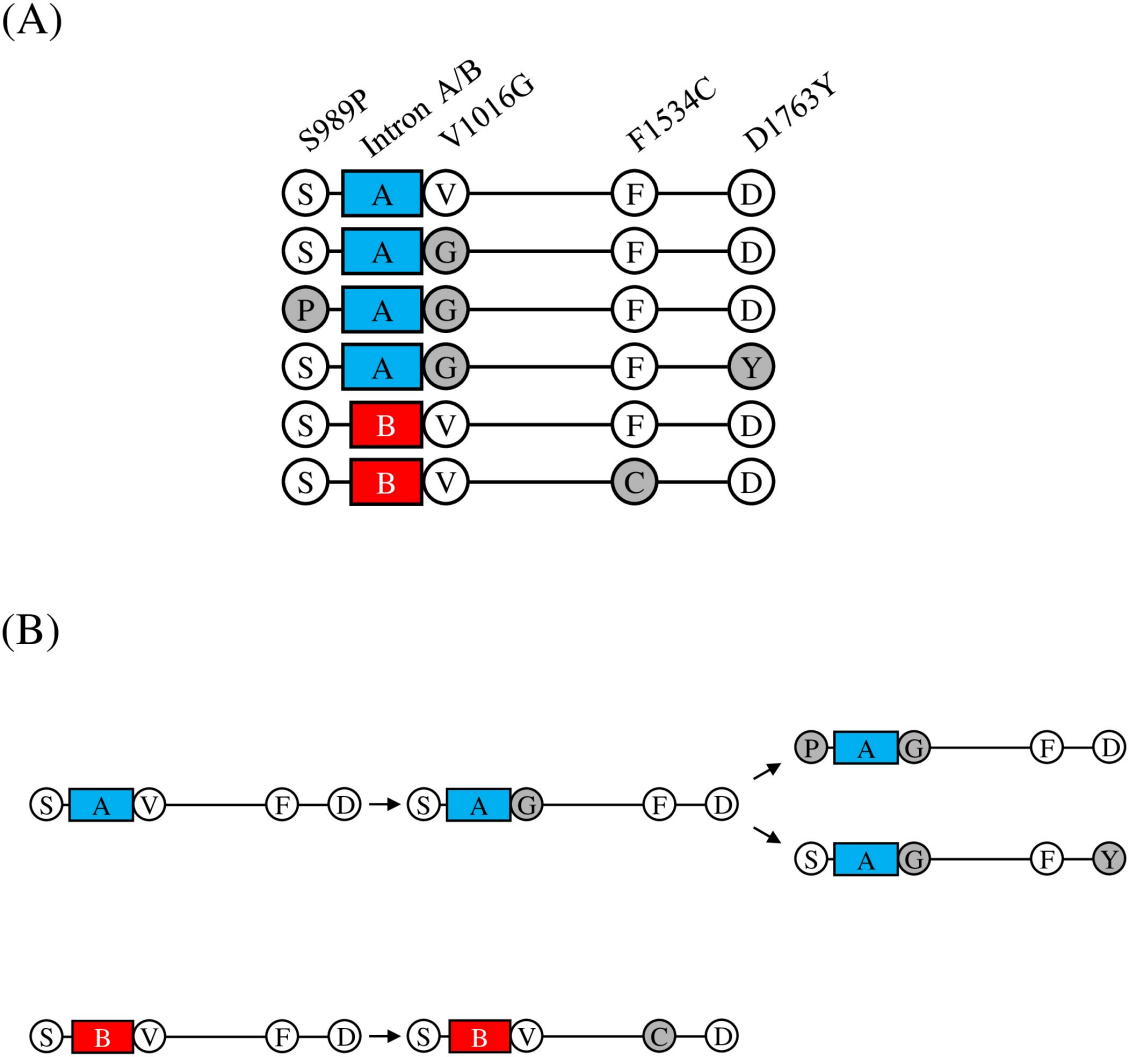
Six VGSC haplotypes in current Taiwanese *Aedes aegypti* (A) and their hypothesized evolutionary pathway (B). White and gray circles represent wild type sites and *kdr* mutations, respectively. Blue and red boxes represent group A and B introns, respectively.

Polymorphism of the intron between exon 20 and 21 of the VGSC gene IIS6 region has been reported in Brazilian *Ae*. *aegypti*. Two forms were classified as group A (250 bp) and B (234 bp) based on their length and sequence differences [14]. In the Taiwanese *Ae*. *aegypti* population, we detected both forms. The genomic position of this intron inserts between V1015 (GTA) and V1016 (GTA). The form belonging to group A is the majority (74.52%) in Taiwanese *Ae*. *aegypti* (Table 1). Remarkably, we noticed that there is a link between VGSC intron polymorphism and *kdr* mutations. In the group A homologous genotype (A/A), we never found F1534C; in the group B homologous genotype (B/B), S989P, V1016G and D1763Y were not found (Fig 3). This phenomenon presumably suggests that S989P, V1016G and D1763Y link the haplotype harboring the group A intron; F1534C then links the group B intron haplotype. According to our description above, we proposed that there should be six VGSC haplotypes in the Taiwanese *Ae*. *aegypti* population (Fig 4A). We identified six VGSC haplotypes from four amino acid positions and polymorphic intron types of two lengths in the genotyped mosquitoes (Fig 4A). Sequencing results were deposited to NCBI GenBank (accession numbers: MK495869~ MK495876). The six haplotypes were deduced from the four existing homozygotes (S989-intron A-V1016-F1534-D1763, S989P-intron A-V1016G-F1534-D1763, S989-intron A-V1016G-F1534-D1763Y and S989-intron B-V1016-F1534C-D1763) and several present heterozygotes (Fig 3). The haplotype number is the smallest that can compatibly reconstitute all the genotypes observed; thus, we proposed six haplotypes in Fig 4, based on the six VGSC haplotype-segregating model. According to the component of these six VGSC haplotypes, we speculated that S989P-intron A-V1016G-F1534-D1763 and S989-intron A-V1016G-F1534-D1763Y were derived from S989-intron A-V1016G-F1534-D1763; S989-intron A-V1016G-F1534-D1763 then originated from S989-intron A-V1016-F1534-D1763. On the other hand, we also proposed that S989-intron B-V1016-F1534C-D1763 was generated from S989-intron B-V1016-F1534-D1763 (Fig 4B). The haplotype harboring group A intron and no mutation (Table 2, S-A-V-F-D) is the majority (46.50%). In both Tainan and Kaohsiung, the proportions of the three haplotypes containing double mutations (S989P+V1016G or V1016G+D1763Y) or a single mutation F1534C (Table 2, including P-A-G-F-D, S-A-G-F-Y and S-B-V-C-D) are higher in October than in March. This phenomenon may be due to pyrethroid insecticide use to control mosquitoes during the summer and autumn in southern Taiwan. Curiously, the proportion of the haplotype containing the single mutation V1016G is higher in March than in October, in both Tainan and Kaohsiung (Table 2, S-A-G-F-D). Mosquitoes caught in October were thought to be more resistant to pyrethroids than those caught in March, owing to the selection by chemical control with pyrethroid insecticides. V1016G plays a critical role in kdr resistance against pyrethroid compounds. The coexistence of S989P was demonstrated to strengthen V1016G-dependent resistance [12]. We speculated that the loss of the V1016G haplotype from March to October possibly resulted from the gain of the two haplotypes harboring V1016G along with either S989P or D1763Y (Table 2, P-A-G-F-D and S-A-G-F-Y). The data support the hypothesis that the coexistence of S989P enhances V1016G-mediated resistance and implies that D1763Y might play a role in V1016G-dependent resistance to cypermethrin.

**Table 2:** Proportion of six haplotypes of the VGSC gene in *Aedes aegypti* collected in southern Taiwan. The sites of the *kdr* mutations are underlined.

Location	Month	Haplotype (989-intron-1016-1534-1763)					
		S-A-V-F-D	S-A-G-F-D	P-A-G-F-D	S-A-G-F-Y	S-B-V-F-D	S-B-V-C-D
Tainan	Mar	70.00%	6.25%	0.00%	5.00%	5.00%	13.75%
		(56/80)	(5/80)	(0/80)	(4/80)	(4/80)	(11/80)
	Oct	51.25%	2.50%	5.00%	10.00%	6.25%	25.00%
		(41/80)	(2/80)	(4/80)	(8/80)	(5/80)	(20/80)
Kaohsiung	Mar	60.81%	6.76%	9.46%	0.00%	2.70%	20.27%
		(45/74)	(5/74)	(7/74)	(0/74)	(2/74)	(15/74)
	Oct	5.00%	0.00%	56.25%	10.00%	0.00%	28.75%
		(4/80)	(0/80)	(45/80)	(8/80)	(0/80)	(23/80)
Total		46.50%	3.82%	17.83%	6.37%	3.50%	21.97%
		(146/314)	(12/314)	(56/314)	(20/314)	(11/314)	(69/314)

### Insecticide resistance to cypermethrin

**Fig 5:**
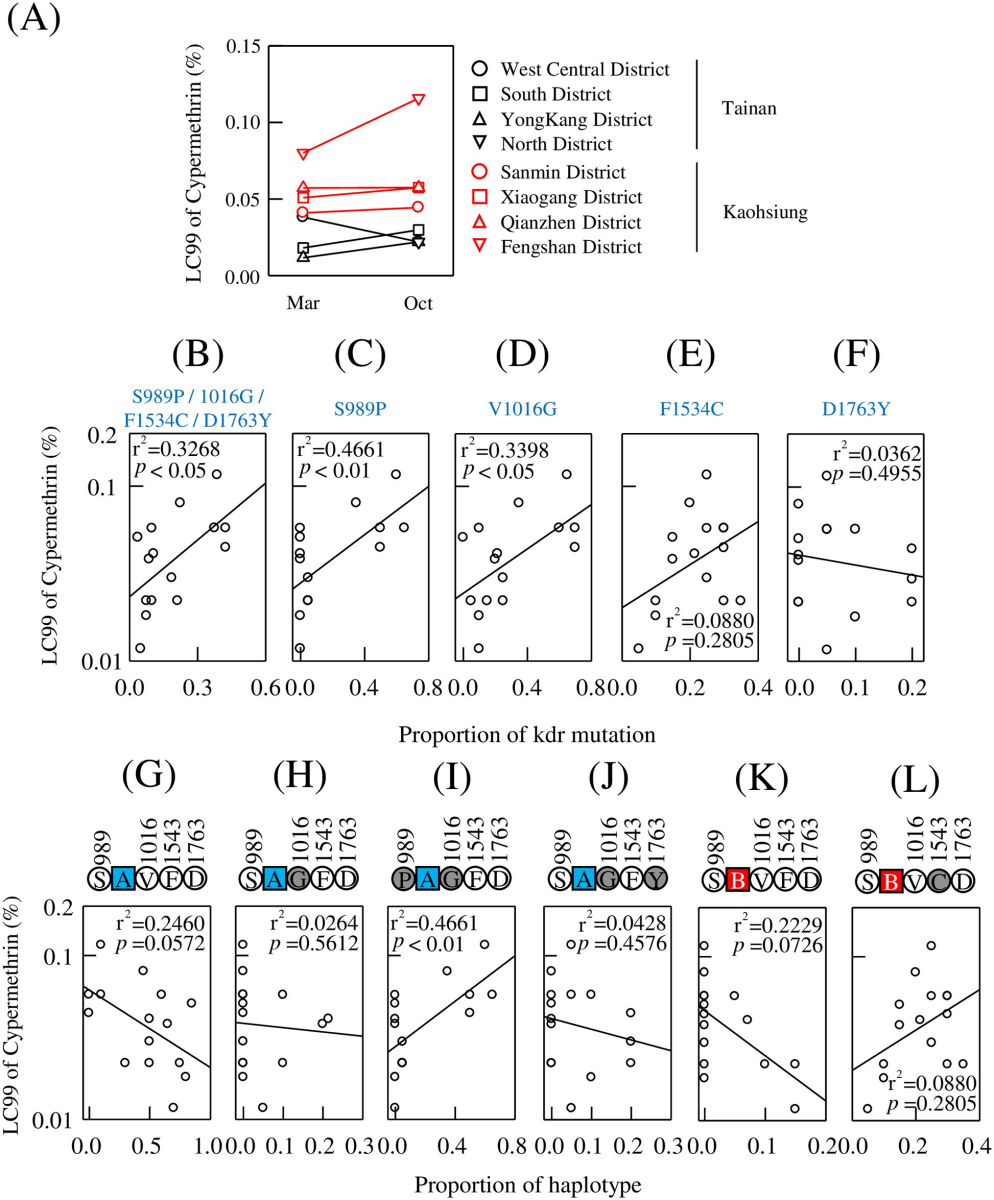
The relationship between cypermethrin resistance and *kdr* mutations. (A) LC99 of cypermethrin in Taiwanese *Ae*. *aegypti* collected in March and October. Mosquitoes from Tainan and Kaohsiung are labeled as black and red plots. (B-F) The relationship between cypermethrin resistance and all four *kdr* mutations (B) or compared with each mutation individually (C-F). (G-L) The relationship between cypermethrin resistance and each VGSC haplotype. The labels of the *kdr* mutations and intron polymorphism are the same as in Fig 4.

Cypermethrin is a widely used pyrethroid insecticide in Taiwan. We examined the mosquitoes’ resistance level to cypermethrin. Fig 5A shows cypermethrin LC99 values for mosquitoes collected from eight districts in Tainan and Kaohsiung. Due to the limited number of insects caught, we could not accomplish a bioassay of mosquitoes collected in March while the North District of Tainan [8 (locations) x 2 (months)—1 (miss) = 15 datasets]. In general, the mosquitoes collected in October are more resistant to cypermethrin than those collected in March, except in West Central District of Tainan. The phenomenon could be interpreted by the heavy insecticide use during summer and autumn in Taiwan. Notably, the mosquitoes collected in Kaohsiung are more resistant to cypermethrin than those collected in Tainan. The temporal and spatial differences in insecticide resistance level also reflect the proportions of VGSC mutations and haplotypes in the genotyped mosquitoes (Fig 3 and Tables 1 and 2). To verify the role of VGSC mutations in cypermethrin resistance in Taiwanese *Ae*. *aegypti* populations, we analyzed the relationship between insecticide resistance level and the proportion of these four mutations in each district from March or October by regression analysis. There is a positive correlation between them (r^2^ = 0.3268, *p* < 0.05) (Fig 5B). We also verified the relation between insecticide resistance level and proportions of each mutation individually. S989P (r^2^ = 0. 4661, *p* < 0.01) and V1016G (r^2^ = 0. 3398, *p* < 0.05) have a higher correlation to cypermethrin LC99 (Fig 5C and 5D), whereas the frequency of mutations at F1534C (r^2^ = 0. 0880, *p* = 0.2805) and D1763Y (r^2^ = 0. 0362, *p* = 0.4955) have lower correlations to the cypermethrin resistance level (Fig 5E and 5F). From another point of view, since we could classify VGSC haplotypes into six categories, we also analyzed the relationship between cypermethrin resistance level and haplotype proportion in different mosquito populations. Not surprisingly, the haplotype with both S989P and V1016G mutations (S989P-intron A-V1016G-F1534-D1763) displays the highest correlation to cypermethrin resistance level with r^2^ = 0.4661 and *p* < 0.01 (Fig 5I), whereas the r^2^ values of the other three haplotypes carrying mutation(s) range from 0.0264 to 0.0880, with *p* values ranging from 0.5612 to 0.2805 (Fig 5H, 5J and 5L). These results suggested that the presence of the two *kdr* mutations, S989P and V1016G, may provide a reference to assess cypermethrin resistance level in Taiwanese *Ae*. *aegypti* populations.

## Discussion

In Taiwan, two *Ae*. *aegypti* VGSC point mutations were reported previously [19, 20]. In those two articles, the authors described the two mutations as V1023G and D1794Y. The amino acid numbers of both were based on the yellow fever mosquito VGSC protein sequence. When the codons are transformed to the house fly VGSC protein sequence, V1023G and D1794Y refer to V1016G and D1763Y, respectively (eight corresponding positions of VGSC mutations between yellow fever mosquito and house fly were clearly annotated in [8]). In the present study, we detected V1016G and D1763Y as well. Moreover, we identified two new VGSC mutations, S989P and F1534C, in Taiwanese *Ae*. *aegypti* populations; however, the causation of these mutations is not clear and requires further in depth research [24]. Our primer sets, following the previous report [23] are also capable of detecting the I1011M/V mutation. In the 157 mosquitoes collected, we found no examples of I1011M/V. The absence of I1011M/V in Taiwanese *Ae*. *aegypti* is not a surprising result. I1011M/V substitutions strictly distribute in the Americas [13, 14, 25–27]. In other regions, the I1011V mutation was only detected in Thailand and Vietnam [28, 29].

Chang *et al*. was the first group to identify *Ae*. *aegypti* VGSC mutations in Taiwan [19]. They sequenced the entire coding region of VGSC genomic DNA from a permethrin resistant strain derived from mosquitoes collected in Kaohsiung in 1990. When compared with two susceptible strains, Bora and NS, the authors found two mutations, V1016G and D1763Y. At that time, V1016G had been identified previously [25], whereas D1763Y was a novel mutation. The permethrin resistant strain had been maintained in the laboratory under constant selection with permethrin for 35 generations [30]. It is of interest whether the novel mutation originated during the pressure of long term permethrin treatment inside the laboratory. In a later work [20], Lin *et al*. surveyed *Ae*. *aegypti* collected from Tainan and Kaohsiung in 2008. From the mosquitoes caught in the field, they indeed could detect D1763Y. Our surveillance of eight districts in Tainan and Kaohsiung demonstrates that D1763Y distributed in all zones as well. Altogether, the data suggest that the mutation D1763Y originated from a wild population in the field but was not a selected product under long-term exposure to permethrin in the laboratory.

Among introns of the VGSC gene, the intron between exons 20 and 21 is polymorphic. Based on their length, these various intron types were classified into group A (250 bp) and B (234 bp) [14]. Intriguingly, the intron polymorphism may serve as a marker to track the origins of *kdr* mutations. I1011M and V1016I were reported to be concurrent with the group A intron in Brazilian *Ae*. *aegypti* [14, 31]. In Africa, F1534C was found to possess a strong link to the group A intron but was rarely coupled with the group B intron [16]. In this paper, we discovered that S989P, V1016G and D1763Y strictly coexist with the group A intron; F1534C was with the group B intron (Figs 3 and 4A). When previous reports [14, 16, 31] and our results are taken together, they suggest that the F1534C *kdr* mutation might originate from multiple historical events, whereas S989P, I1011M, V1016G/I and D1763Y might individually come from one single occurrence. Curiously, the etiology of most *Ae*. *aegypti* VGSC mutations being exclusively concurrent with the group A intron remains an interesting puzzle to verify. In our results and previous investigations [14, 16], alleles harboring the group A intron are the majority in the population. We speculated that this may be because most *kdr* mutations are located on alleles with group A intron, since yellow fever mosquitoes in the world are constantly under the pressure of pyrethroid insecticides. The selection force presumably keeps the alleles carrying *kdr* mutations.

In our results, all S989P and D1763Y mutations are concurrent with V1016G (Figs 3 and 4A). The coexistence of S989P and V1016G was reported previously [32, 33], as well as D1763Y and V1016G [19]. The combination of the membrane protein of site-directed mutagenesis expressed in *Xenopus* oocytes and the two electrode voltage clamp technique constructs a platform to examine the function of the VGSC mutation [9]. V1016G was found to reduce the sensitivity of expressed protein to permethrin and deltamethrin [11, 12]. S989P did not alter the sensitivity of recombination protein to permethrin and deltamethrin [11, 12], nor did D1763Y [11]. It is of great interest to examine whether a *kdr* mutation can assist another mutations resistance to pyrethroid, particularly when a mutation, such as S989P or D1763Y, needs to be concurrent with V1016G rather than alone. When S989P was cointroduced with V1016G, S989P did not change the response of V1016G to permethrin [11, 12], but reduced the sensitivity to deltamethrin [12]. D1763Y was coupled with V1016G in the permethrin resistant strain [19], implying that D1763Y might confer an assistant role to enforce or strengthen V1016G’s resistance to permethrin. Indeed, we observed an increasing proportion of the haplotype harboring V1016G and D1763Y from March to October (Table 2), strong evidence that D1763Y was involved in V1016G-dependent resistance to cypermethrin. However, the coexpression of D1763Y could not alter V1016G’s resistance to permethrin and deltamethrin [11]. The function of D1763Y in pyrethroid resistance remains to be further investigated.

The association between VGSC haplotype and pyrethroid resistance was clearly demonstrated in this study. The haplotype harboring both S989P and V1016G was positively correlated with Pyrethroid resistance (Fig 5I), which is concordance with the study of Kasai *et al*. [23]. They revealed the reduced susceptibility accompany with the increased frequency of S989P and V1016G after repeated pyrethroid selection in the laboratory. It is not surprisingly to see no other haplotypes had significantly correlation with insecticide resistance alone. However, the VGSC genotype is comprised of 2 haplotypes in one mosquito; the role of VGSC genotypes in pyrethroid resistance deserves further understanding.

In our results, in the mosquito groups with stronger resistance to cypermethrin, more S989P and V1016G are present. This phenomenon was supported by both views of the VGSC mutation proportions of either S989P or V1016G (Fig 5C and 5D) and the S989P+V1016G haplotype (Fig 5I). Being aware of pests’ resistance level to insecticides will be helpful in pest control strategy. However, the bioassay to probe certain population’s resistance level to insecticides requires numerous live mosquitoes and is usually time-consuming. For the areas that need to use cypermethrin to control *Ae*. *aegypti*, our data may propose an alternative method where the proportion of S989P and V1016G in the population perhaps can serve as a reference to estimate the cypermethrin resistance level.

The complexity of genetic components allows organisms to survive through various challenges during natural selection. The *kdr* mutations in the *Ae*. *aegypti* VGSC gene play a vital role to help mosquitoes resist the disturbance of pyrethroid molecules targeting the neural VGSC protein [8–10]. The accumulation of *kdr* mutation types may benefit insect fitness to resist pyrethroid insecticides. After Bregues *et al*. initially identified VGSC mutations from strains resistant to pyrethroid and DDT [25], to date at least ten mutations have been reported. In various regions around the world, more than one mutation in certain mosquito populations was widely recorded [8, 10]. More recently, the coexistence of three mutations (S989P, V1016G and F1534C) was reported from Southeast Asia [34–37]. In this paper, we reported that currently there are four *kdr* mutations, namely S989P, V1016G, F1534C and D1763Y, in *Ae*. *aegypti* populations in Taiwan. These four mutations likely would be an obstacle to the control and prevention of diseases transmitted by *Ae*. *aegypti*. In summary, the present study is the first article to report the coexistence of four *kdr* mutations in a population.
